# Understanding potential implications for non-trophic parasite transmission based on vertebrate behavior at mesocarnivore carcass sites

**DOI:** 10.1007/s11259-021-09806-2

**Published:** 2021-06-26

**Authors:** Moisés Gonzálvez, Carlos Martínez-Carrasco, Marcos Moleón

**Affiliations:** 1 Department of Animal Health, Faculty of Veterinary Sciences, Regional Campus of International Excellence “Campus Mare Nostrum”, University of Murcia, 30100 Murcia, Spain; 2 Department of Zoology, Faculty of Sciences, University of Granada, 18071 Granada, Spain

**Keywords:** Carnivore, Carrion, Non-trophically transmitted parasites, *Sarcoptes scabiei*, Scavenger, Wildlife

## Abstract

High infection risk is often associated with aggregations of animals around attractive resources. Here, we explore the behavior of potential hosts of non-trophically transmitted parasites at mesocarnivore carcass sites. We used videos recorded by camera traps at 56 red fox (*Vulpes vulpes*) carcasses and 10 carcasses of other wild carnivore species in three areas of southeastern Spain. Scavenging species, especially wild canids, mustelids and viverrids, showed more frequent rubbing behavior at carcass sites than non-scavenging and domestic species, suggesting that they could be exposed to a higher potential infection risk. The red fox was the species that most frequently contacted carcasses and marked and rubbed carcass sites. Foxes contacted heterospecific carcasses more frequently and earlier than conspecific ones and, when close contact occurred, it was more likely to be observed at heterospecific carcasses. This suggests that foxes avoid contact with the type of carcass and time period that have the greatest risk as a source of parasites. Overall, non-trophic behaviors of higher infection risk were mainly associated with visitor-carcass contact and visitor contact with feces and urine, rather than direct contact between visitors. Moreover, contact events between scavengers and carnivore carcasses were far more frequent than consumption events, which suggests that scavenger behavior is more constrained by the risk of acquiring meat-borne parasites than non-trophically transmitted parasites. This study contributes to filling key gaps in understanding the role of carrion in the landscape of disgust, which may be especially relevant in the current global context of emerging and re-emerging pathogens.

**Graphical abstract:**

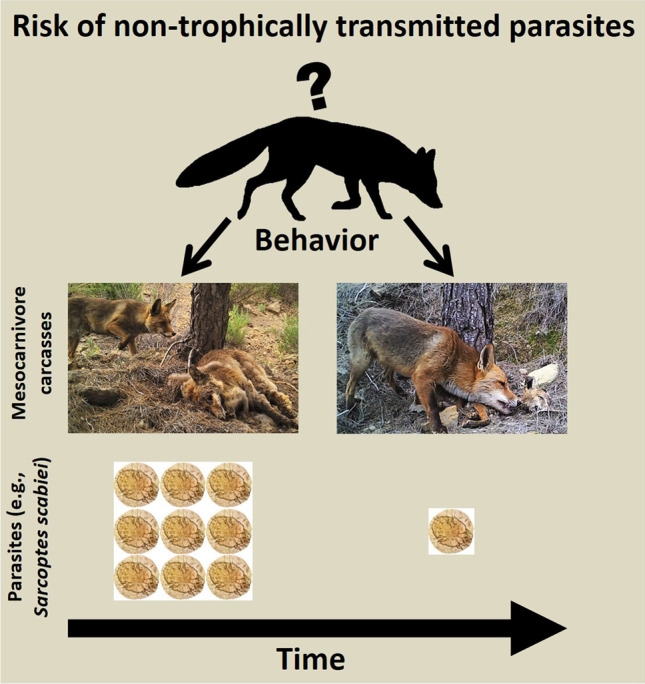

**Supplementary Information:**

The online version contains supplementary material available at 10.1007/s11259-021-09806-2.

## Introduction

Pathogen distribution is spatially and temporally heterogeneous, so epidemiological landscapes frequently consist of hotspots for transmission risk within a matrix of area with reduced or even no exposure to parasites (Bousema et al. 2012; Buck et al. 2018; Weinstein et al. 2018a). Infection risk hotspots may be driven by the presence of attractive resources that favor aggregation of animals, such as water points and food-rich patches, or by specific sites where pathogens are more like to accumulate, such as latrines (Buck et al. 2018; Weinstein et al. 2018a, b). Epidemiological risk may also be increased by species-specific behaviors, such as social interactions between individuals of gregarious species or family groups, or during certain times of year, such as the rutting season (Altizer et al. 2003; Patterson and Ruckstuhl 2013; Ezenwa et al. 2016). Infection risk also depends on the diversity of susceptible and alternative hosts in the environment (Johnson and Thieltges 2010). In this context, when food resources and other points of attraction are apparently infected, hosts must weigh the perceived infection risk against foraging gains and other benefits (Weinstein et al. 2018b). Understanding host behavioral responses to potential risk of infection associated with food resources is relevant from an ecological and evolutionary perspective, but also provides a solid basis for better interpreting the epidemiological risk factors that favor the transmission of pathogens in the wild (Hart 1990; Kuris 2003; Penczykowski et al. 2015; Stockmaier et al. 2021).

Carcasses are a paradigmatic example of a food resource that may be regarded as hotspots for both trophically and non-trophically transmitted pathogens (Turner et al. 2014; Dmitric et al. 2017; Moleón and Sánchez-Zapata 2021). This nutrient-rich resource attracts many scavengers in all ecosystems (DeVault et al. 2003; Beasley et al. 2012; Mateo-Tomás et al. 2015; Sebastián-González et al. 2019), leading to concentrations around carcasses of up to hundreds of individuals in the case of vultures (Donázar 1993). In the absence of vultures, which are very efficient carrion consumers, many opportunistic or facultative scavengers, such as mammalian mesocarnivores, may readily access carrion (Morales-Reyes et al. 2017). In these conditions, parasite transmission may occur not only from the carcass to the scavenger (Byrom et al. 2015; Straub et al. 2015), but also among different scavengers that co-occur at carcass sites (Ogada et al. 2012; Borchering et al. 2017). Moreover, the dead animal can be a source of pathogens for non-scavenging species that approach the carcass without the intention of eating it, for other species that contact the carcass with the aim of ingesting the necrophagous invertebrates found on it, as well as for animal species that use the carcass for non-trophic purposes, such as marking behavior and taking material for nest construction (Moleón and Sánchez-Zapata 2016, 2021).

Carcasses are normally an ephemeral resource (DeVault et al. 2003; Barton et al. 2013). However, not all of them have the same duration in the environment. Carcasses of carnivorous species generally persist longer than those of herbivorous species (Selva et al. 2005; Olson et al. 2016; Moleón et al. 2017, 2020). Field observations indicate that carnivorous species avoid feeding on carcasses of phylogenetically related species, especially on conspecific carcasses, probably due to the increased risk of acquiring species-specific meat-borne parasites (Hart 2011; Moleón et al. 2017). Therefore, the opportunities for contact between carcasses and the visiting vertebrate species, as well as between the latter, are higher in the case of carnivore carcasses. Consequently, the possibility that visiting species may be infected through this type of carcass, even if not consumed, may also increase. Thus, carnivore carcasses are an excellent model to study host behavior around carcasses and how this behavior changes with time; in this way, it could be inferred whether this behavior carries a risk of acquiring non-trophically transmitted parasites. However, fine-grained behavioral studies about the risk associated with carcass sites are largely lacking, particularly for carnivore carrion (Moleón and Sánchez-Zapata 2021).

In the case of mammalian carnivores, non-trophically transmitted pathogens include a wide range of parasites, fungus, bacteria and viruses. These pathogens have characteristics that largely condition their virulence and transmission, such as survival time in the environment of the infective stages, within-host replication rate, pathogen infectivity, the route of infection, the number of host species that are susceptible, and the life cycle they present (Poulin 2007; Alizon and Michalakis 2015; Acevedo et al. 2019; Brouwer et al. 2019). The persistence outside the host of infective stages can vary from a few hours to many years, depending on pathogen characteristics and environmental factors (Traversa et al. 2014; Chenais et al. 2018). With regard to carcasses, it is assumed that, in general terms, the number of infective forms and their survival decreases as the distance to the carcass site increases and over time, although few studies have investigated this topic (Turnbull et al. 1998; Fialho et al. 2018; Rossi et al. 2019).

Among the non-trophically transmitted pathogens that cause the greatest impact on wildlife is the mite *Sarcoptes scabiei*, an obligate permanent parasite that causes sarcoptic mange (Niedringhaus et al. 2019). This multi-host ectoparasite is widely distributed and affects a broad range of mammals, including ungulates and carnivores (Carricondo-Sánchez et al. 2017; Pisano et al. 2019; Turchetto et al. 2020). These mites live in the epidermis of their hosts, and can be transmitted through direct contact between animals or indirectly when a susceptible host acquires free mites that have shed the skin of an infected animal, especially in dens and other sheltered sites where *S. scabiei* may survive for several days (Pence and Ueckerman 2002). Another infectious agent of major concern, due to its health impact on wildlife populations, is the bacterium *Bacillus anthracis*, which causes anthrax in ungulates and, to a lesser extent, in carnivores (Hugh-Jones and de Vos 2002). After the death of the infected animal, this virulent pathogen produces spores around the carcass that can persist in the environment for years, infecting new hosts via ingestion or inhalation (Bellan et al. 2013; Turner et al. 2014). Other widely distributed, non-trophically transmitted infectious agents that can seriously affect wild carnivore populations are rabies, distemper virus and canine parvovirus, which can be acquired through the saliva, respiratory secretions and feces of infected animals, respectively (Truyen et al. 1998; Nouvellet et al. 2013; McElhinney et al. 2014).

One of the paradigmatic hosts of these pathogens is the red fox (*Vulpes vulpes*), the most broadly distributed mammalian carnivore worldwide. This generalist species feeds upon a wide array of trophic resources, including vertebrate and invertebrate prey, plants, fungi and carrion (Wilson and Mittermeier 2009; Mateo-Tomás et al. 2015). Foxes occupy a wide range of habitats, including urban and peri-urban areas (Wilson and Mittermeier 2009). The ubiquity and ecological plasticity of foxes has led to recurrent scientific discussions about their epidemiological role in the maintenance and dispersion of pathogens with potential zoonotic and veterinary significance (Di Cerbo et al. 2008; Karamon et al. 2018).

Our main goal is to explore the behavior of potential hosts of non-trophically transmitted pathogens at carnivore carcass sites, with a special emphasis on the red fox. For this purpose, we monitored the decomposition process of fox and other mesocarnivore carcasses in several areas that differ in their communities of vertebrate carnivores and levels of anthropization. Analyzed behaviors include direct contact, marking and rubbing, either on the carcass or in its vicinities. Our main hypothesis is that the risk of acquiring pathogens through direct contact is dependent on both time since the carcass became available and carcass type (conspecific *vs*. heterospecific regarding the consumer), and that hosts rely on indirect cues to shape their behavior at carcass sites. Overall, we predict that risky behaviors will be more frequent at late stages of carcass decomposition and in heterospecific carcasses. This study may provide important insights to further understand the landscape of disgust associated with carrion, as well as the possible epidemiological consequences of this host behavior (Buck et al. 2018; Weinstein et al. 2018a; Doherty and Ruehle 2020; Moleón and Sánchez-Zapata 2021). This kind of study may be especially relevant in the current SARS-CoV-2 pandemic context, which has highlighted the need to investigate the forms of transmission of this emerging pathogen (Wong et al. 2020) in wild species, especially in mesocarnivores (Leroy et al. 2020; Tiwari et al. 2020).

## Material & methods

### Study areas

Fieldwork was carried out in three mountainous areas of southeastern Spain: Sierras de Cazorla, Segura y Las Villas Natural Park (hereafter Cazorla; 2,099 km^2^, 38º09’N 2º44’W), Sierra Espuña Regional Park (hereafter Espuña; 178 km^2^, 37º51’N 1º32’W) and periurban areas of Murcia city (hereafter Murcia; 415 km^2^, 37º57’N 1º02’W). Natural vegetation in these three areas is dominated by pine forests (mostly *Pinus halepensis* at low altitudes and *P. nigra* and *P. pinaster* at higher altitudes), aromatic shrubs, and patches of oak forests (*Quercus ilex* and *Q. faginea*) (Rivas-Martínez 1987). There is an altitudinal and meteorological gradient from Cazorla (500–2,107 m a.s.l.; mean annual temperature: 12-16ºC; mean annual precipitation: 300–950 mm) to Espuña (200–1,583 m a.s.l.; 13-18ºC; 300–500 mm) and Murcia (190–490 m a.s.l.; 17-23ºC; 200–450 mm) (www.juntadeandalucia.es; siam.imida.es). Meso-, Supra- and Oro-Mediterranean stages are represented in Cazorla, Thermo-, Meso- and Supra-Mediterranean stages in Espuña, and Thermo- and Meso-Mediterranean stages in Murcia (Rivas-Martínez 1987). Cazorla and Espuña are protected areas, while Murcia supports moderate to high levels of anthropization, including scattered residential areas and herbaceous and fruit tree cultivations (mainly citrus trees).

In general, vertebrate communities are much richer in Cazorla, which holds a large resident population of obligate scavengers (i.e., vultures) and a wide variety of facultative scavengers. The scavenging community is similar in Espuña, though vultures are less abundant. In Murcia, vultures are mostly absent, and domestic carnivores, such as the dog (*Canis lupus familiaris*) and the cat (*Felis silvestris catus*), are more frequent. The fox is the commonest wild mammalian carnivore in the three study areas, though it is more abundant in Espuña than in Cazorla; there are no detailed data for Murcia. For more information on Cazorla and Espuña, see Moleón et al. (2017) and Morales-Reyes et al. (2017).

### Data collection

A total of 66 mesocarnivore carcasses were monitored in Cazorla (n = 27 foxes), Murcia (n = 19 foxes) and Espuña (n = 20 carcasses, including ten foxes, four stone martens *Martes foina*, three Eurasian badgers *Meles meles*, two common genets *Genetta genetta* and one wildcat *Felis silvestris silvestris*) from November 2016 to March 2018. The main research model was the fox because it is the most abundant carnivore in the studied areas. Hereafter, carcasses of carnivores other than foxes are designated as “other carcasses”. Carcasses came from authorized hunting (only in the case of foxes) and recent road kills (foxes and other carnivores). Immediately after collection, carcasses were eviscerated, and a serum sample was taken from each animal to perform enzyme-linked immunosorbent assays for antibody detection (ELISA kits, Ingenasa®, Madrid, Spain) against some infectious diseases (canine distemper virus CDV, feline coronavirus FCoV, canine and feline parvovirus CPV/FPV, feline leukemia virus FeLV and feline immunodeficiency virus FIV). In addition, muscle samples from the base of the tongue, the forearms and diaphragm were processed by artificial digestion to detect the presence of *Trichinella* spp. larvae (Kapel et al. 1994; Gamble et al. 2000). Carcasses used in the study were free from these pathogens, and no lesions compatible with sarcoptic mange, mycosis or other pathologies were detected. In the case of hunted foxes, the tissues adjacent to the shot were removed to eliminate any trace of lead. After necropsy, carcasses were frozen at -20 °C in individual plastic bags, with the time elapsed between carnivore death and freezing being less than 18 h (Moleón et al. 2017).

Carcasses were defrosted before their placement in the field for 12–24 h at room temperature. Carcasses were randomly distributed throughout the study areas, with a minimum distance between neighboring cameras of at least 1 km (Moleón et al. 2017). Each carcass was fixed to a rock or a tree trunk with 1.5 mm diameter steel wires to avoid movement of the carcasses by scavengers away from the recording field of the camera. The wires were camouflaged with plants and soil (Moleón et al. 2015). Altitudinal range for carcass sites was 772–1676 m a.s.l. in Cazorla, 433–1432 m a.s.l. in Espuña and 125–448 m a.s.l. in Murcia. On the micro-habitat scale (i.e., radius of 10 m around the carcass), sampling places were categorized as “close areas”, when the vertical projection of trees and shrubs exceeded 50%, and “open areas” otherwise.

Carcasses were monitored using automatic cameras (Bushnell Trophy Cam and Bushnell Aggressor) until complete consumption (i.e., no remains, or only fur left) or for a maximum of 10 weeks if the carcass was not completely consumed (i.e., bones and skin remained). Cameras were placed in discreet locations close to the carcasses (3–4 m) and were programmed to record a 15-s video every minute when detecting movement. Each carcass site was visited weekly to check batteries and memory cards. Cameras provided information on the presence of vertebrate species and their behavior at carcass sites. Recorded vertebrate species were classified into three groups: “red fox”, “other mammals” and “birds” (the reptile *Timon lepidus* was also included in this last group, due to the scarce number of events in which this species was recorded). Based on O’Brien et al. (2003) and Ridout and Linkie (2009), we defined independent events for each carcass as: a) consecutive videos of unequivocally different individuals of the same species or individuals of different species; b) when individual identification was not possible, consecutive videos of individuals of the same species taken more than 30 min apart; and c) non-consecutive videos of individuals of the same species. For each event, we recorded a) the species group, b) the number of different individuals, c) the existence of direct contact between the visitor and the carcass, d) the existence of marking behavior (urine and feces deposition), e) the existence of rubbing behavior, and f) the minimum distance between the visitor and the carcass (“contact”: distance = 0 cm; “close”: distance > 0–50 cm; “moderate”: distance > 50–200 cm; “far”: distance > 200 cm). These distance intervals were also used to classify marking and rubbing sites.

### Data analyses: weekly behavioral patterns of species visiting the carcasses

We explored the general spatiotemporal patterns of mesocarnivore carcass use by the studied vertebrate communities. First, for each study area and carcass type (foxes and others), we calculated, on a weekly basis, the proportion of carcasses that were contacted (i.e., with at least one direct contact event), marked (i.e., with at least one marking behavior event), rubbed (i.e., with at least one rubbing behavior event on the carcass or on the ground next to it), and visited but not contacted (i.e., no contact events recorded), for all vertebrates together and separately for each vertebrate group. For each study area and carcass type, we also estimated the number of contact, marking, rubbing and no contact events per carcass. Second, we calculated the accumulated number of carcasses that were a) detected, b) contacted (i.e., at least one contact event), c) marked (i.e., at least one marking event), and d) rubbed (i.e., at least one rubbing event) each week by foxes.

### Data analyses: determinants of fox behavior

We used generalized linear models (GLMs) to analyze the factors influencing “time of first contact” (only carcasses with at least one contact event by foxes were used; n = 54). We conducted two separate analyses, using these two different datasets: 1) all fox carcasses in the three study areas; and 2) both fox and other carcasses in Espuña only. The first analysis is mainly aimed at exploring the general behavior of foxes at conspecific carcasses, while the second is aimed at determining if fox behavior is influenced by carcass type. Time of first contact was calculated as the time elapsed since carcass detection by foxes until the first contact event by foxes. The sample unit for these analyses was the carcass. The explanatory variables for the first analysis were study “area” (Cazorla, Espuña, Murcia), “habitat” (close, open), “year”, “season” (winter: November-February; spring: March and April), “hour” of carcass placement (morning: from dawn to 12:00 h; afternoon: from 12:00 h to dusk), and carcass “detection time” by foxes (i.e., time elapsed since carcass placement and its detection by fox, expressed in days). The explanatory variables for the second analysis were “carcass type” (fox, other) and carcass “detection time” by foxes.

We then ran univariate models (Gaussian error distributions and identity functions) with all the possible explanatory variables for each case. Model selection was based on Akaike’s Information Criterion, which allows the identification of the most parsimonious model (lowest AIC) and ranks the remaining models. For each model, the AIC value was corrected for small sample sizes (AICc). Then, delta AICc (ΔAICc) was calculated as the difference in AICc between each model and the best model in the evaluated set, and models with ΔAICc < 2 were considered to have similar support (Burnham and Anderson 2002). We calculated the deviance (D^2^) explained by each candidate model according to this formula: D^2^ = (null deviance – residual deviance) / null deviance *100 (Burnham and Anderson 2002).

Finally, we used Chi-square analyses to compare the minimum distance between visiting foxes and the carcass a) among study areas (only fox carcasses) and b) carcass types (only in Espuña). All analyses were done with R Studio software v1.0.143 (R Core Team 2018).

## Results

### Visiting species

We recorded a total of 2,383 events (58.9% in Cazorla, 23.9% in Murcia, 7.9% in Espuña at fox carcasses, 9.3% in Espuña at other carcasses) of 41 vertebrate species (19 birds, 21 mammals and one reptile) visiting the carcasses. The average richness of visiting species per carcass in Cazorla was approximately double that in Murcia and Espuña (Tables S1 and S2). Domestic species (mainly dogs, but also cats, goats and sheep) were rarely recorded (1.4% of total events; Table S2). The fox was the most frequently recorded species in the three study areas (40.3% of total events), followed by European robin (*Erithacus rubecula*; 8.9%), wild boar (*Sus scrofa*; (7.0%), Eurasian jay (*Garrulus glandarius*; 6.6%), carrion crow (*Corvus corone*; 4.3%) and stone marten (*Martes foina*; 4.2%), among others. Mean number of different individuals per event was 1.1 ± 0.9 (range: 1–29), and groups of visitors (i.e., more than one individual) were recorded at 8.0% of total events. Groups were more frequently recorded for carrion crow (*Corvus corone*), wild boar (*Sus scrofa*), mouflon (*Ovis aries musimon*) and Eurasian jay (*Garrulus glandarius*) in Cazorla. The fox was very rarely observed in groups (Table S2).

### General patterns of contact, marking and rubbing behaviors

**Fig. 1:**
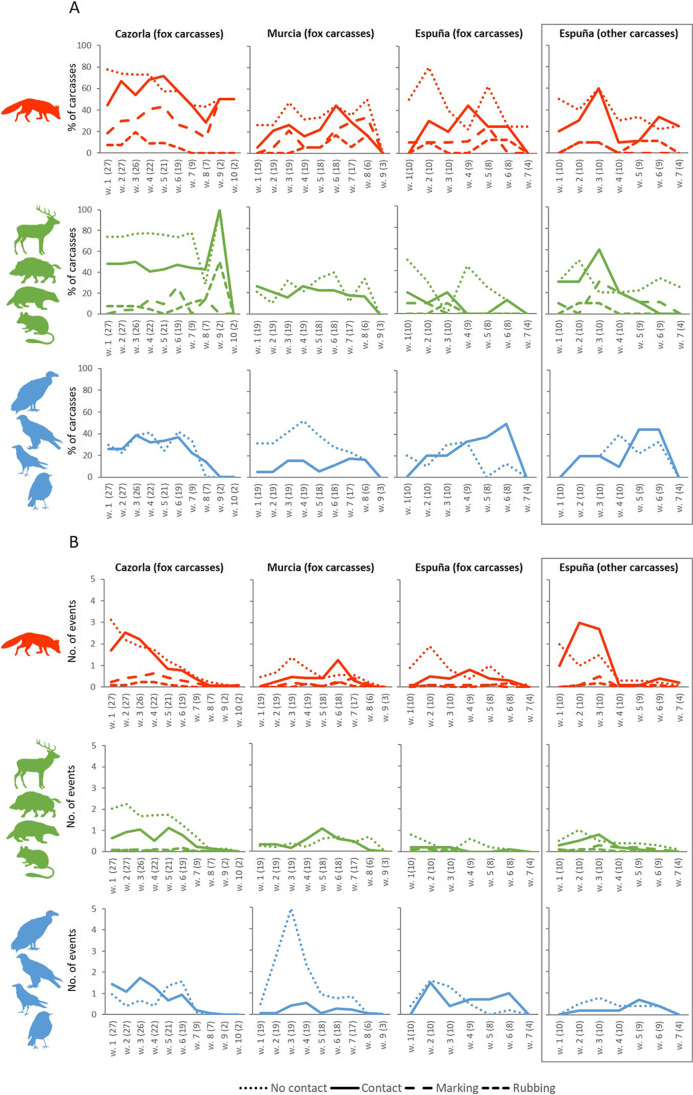
Weekly variation in patterns of use of mesocarnivore carcasses by red fox, other mammals and birds in three areas of southeastern Spain. A) Weekly percentage of contacted (i.e., with at least one contact event), non-contacted (i.e., visited, but no contact events recorded), marked (i.e., with at least one marking event), and rubbed (i.e., with at least one rubbing event) carcasses by red fox, other mammals and birds per study area and carcass type. B) Weekly number of contact, non-contact, marking, and rubbing events by red fox, other mammals and birds per study area and carcass type. For a given week, the number of events is divided by the total number of carcasses studied in each study area, and the number of carcasses available is given in parentheses. Panels for carcasses of carnivores other than foxes are in boxes

Contact events represented 40.6% of the total recorded events (Fig. 1, Table 1). Considering all study areas together, the fox was the species that most frequently contacted carcasses (45.0% of total contact events; Fig. 1, Tables 1 and S2). Intraspecific contact was recorded at 100% of carcasses in Cazorla, 63.2% in Murcia, and 60.0% (fox carcasses) and 30.0% (other carcasses) in Espuña. In foxes, intraspecific contact was detected in 43.4% of the total events recorded. In Espuña, events (especially contact events) of foxes and other mammals, but not of birds, were more frequently recorded at carcasses of other mesocarnivores (Fig. 1). Contact of both domestic and wild species with the same carcass took place at six carcasses in Cazorla (22.2% of total carcasses in this area), three in Murcia (15.8%) and two carcasses of other mesocarnivores in Espuña (20.0% of total non-fox carcasses). Contact between individuals of different visiting species at carcass sites was recorded only once, between a golden eagle (*Aquila chrysaetos*) and a griffon vulture (*Gyps fulvus*) in Cazorla. Consumption by scavengers was recorded at 77.8% of carcasses in Cazorla, 31.6% in Murcia, and 50% and 60% at fox and other carcasses in Espuña, respectively. These trophic behaviors involved 15.7% of total recorded events (Gonzálvez 2020).

**Table 1:** Carcass use patterns per study area and carcass type, according to different vertebrate species groups

Area	Carcass type	N	Group	Carcasses visited	Carcasses contacted	Carcasses marked	Carcasses rubbed	Total events	Contact events	Marking events	Rubbing events
Cazorla	Foxes	27	Red fox	27 (100%)	27 (100%)	20 (74.1%)	9 (33.3%)	22.0 ± 13.8	10.2 ± 9.0	2.8 ± 4.1	0.7 ± 1.6
			Other mammals	27 (100%)	23 (85.2%)	9 (33.3%)	8 (29.7%)	17.0 ± 9.6	1 ± 5.0	0.4 ± 0.8	0.5 ± 0.9
			Birds	21 (77.8%)	18 (66.7%)	0 (0%)	0 (0%)	12.9 ± 15.6	7.4 ± 10.3	0	0
			Total	27 (100%)	27 (100%)	22 (81.5%)	14 (51.2%)	51.9 ± 25.6	23.1 ± 16.7	3.3 ± 4.6	1.2 ± 1.9
Murcia	Foxes	19	Red fox	16 (84.2%)	12 (63.2%)	9 (47.4%)	4 (14.8%)	8.4 ± 8.8	3.3 ± 3.9	1.2 ± 1.6	0.6 ± 1.3
			Other mammals	16 (84.2%)	9 (47.4%)	0 (0%)	0 (0%)	6.9 ± 10.6	3.5 ± 8.1	0	0
			Birds	15 (78.9%)	9 (47.4%)	0 (0%)	0 (0%)	14.6 ± 17.3	1.6 ± 2.6	0	0
			Total	19 (100%)	16 (84.2%)	9 (47.4%)	4 (14.8%)	30.0 ± 25.2	8.4 ± 9.0	1.2 ± 1.6	0.6 ± 1.3
Espuña	Foxes	10	Red fox	9 (90.0%)	6 (60.0%)	4 (40.0%)	1 (10.0%)	7.7 ± 6.2	2.4 ± 3.5	0.9 ± 1.5	0.4 ± 1.3
			Other mammals	10 (100%)	6 (60.0%)	2 (20.0%)	1 (10.0%)	2.8 ± 2.4	0.7 ± 0.7	0.2 ± 0.4	0.1 ± 0.3
			Birds	8 (80.0%)	6 (60.0%)	0 (0%)	0 (0%)	8.3 ± 15.2	4.3 ± 6.7	0	0
			Total	10 (100%)	10 (100%)	5 (50.0%)	2 (20.0%)	18.8 ± 18.4	7.4 ± 8.7	1.1 ± 1.6	0.5 ± 1.3
	Other	10	Red fox	10 (100%)	9 (90.0%)	1 (10.0%)	2 (20.0%)	12.9 ± 24.1	7.5 ± 16.8	0.6 ± 1.9	0.5 ± 1.1
			Other mammals	10 (10.0%)	8 (80.0%)	5 (50.0%)	2 (20.0%)	5.1 ± 4.3	1.9 ± 1.6	0.9 ± 1.3	0.2 ± 0.4
			Birds	7 (70.0%)	5 (50.0%)	0 (0%)	0 (0%)	4.2 ± 4.9	1.7 ± 2.2	0	0
			Total	10 (100%)	10 (100%)	6 (60.0%)	3 (30.0%)	22.2 ± 22.4	11.1 ± 16.1	1.5 ± 2.0	0.7 ± 1.3

Number of monitored carcasses is indicated for each study area and carcass type. The number of carcasses visited, contacted, marked and rubbed by each vertebrate group is shown together with the percentage relative to the total carcasses monitored per area and carcass type (in parentheses). Mean number of events per carcass ± SD is shown for total, contact, marking and rubbing events. We considered carcasses contacted, marked and rubbed as those carcasses with at least one event with contact, marking or rubbing by a given vertebrate group. Similarly, we considered contact, marking and rubbing events as those events with at least one contact, marking or rubbing behavior recorded

Marking and rubbing behaviors were recorded in 5.7% and 2.4% of total events, respectively (Table 1, Fig. 1). Most marking (62.8%) and rubbing (82.5%) events involved direct contact with the carcass. The fox was the most frequently recorded species marking (83.1% of total marking events) and rubbing on the carcass or on the adjacent ground (70.1% of total rubbing events). No marking or rubbing behaviors were observed for birds (Table 1, Fig. 1). Regarding total marking events, urination was more frequently recorded than defecation in foxes (85.2% of total marking events) and other mammals (73.9%).

### Weekly patterns in fox behavior

Carcasses in all the study areas were detected by foxes from the first week. The number of red fox contact events peaked in the second to sixth week in the case of fox carcasses in all areas. In Espuña, the peak for other carcasses took place in the second week, i.e., two weeks earlier than the peak for fox carcasses in this area. While the first contacts with fox carcasses in Cazorla and Murcia, and with other carcasses in Espuña, were recorded in the first week after their deployment, the first events of contact with fox carcasses in Espuña were detected in the second week. In Espuña, foxes contacted more heterospecific carcasses than conspecific ones (Table 1, Fig. 1).

**Fig. 2:**
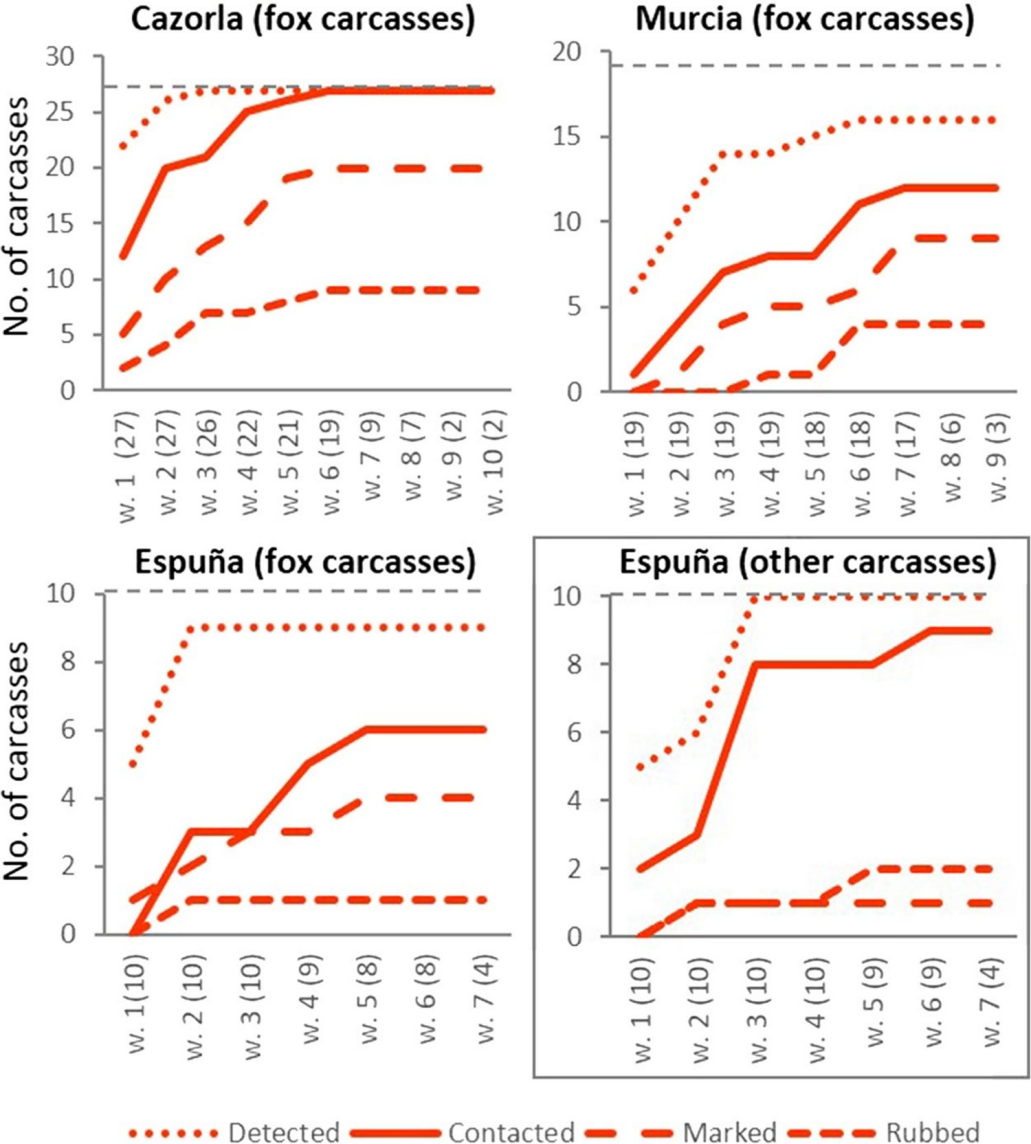
Accumulated weekly number of detected (i.e., with at least one event recorded), contacted (i.e., with at least one contact event), marked (i.e., with at least one marking event), and rubbed (i.e., with at least one rubbing event) carcasses by the red fox per study area and carcass type. Dotted horizontal gray lines represent the accumulated number of available carcasses. For a given week, the number of carcasses available is given in parentheses. Panel for carcasses of carnivores other than foxes is in the box

The accumulated number of fox carcasses contacted by fox ranged between 100% in Cazorla to 60% in Espuña; in the latter area, foxes contacted 90% of carcasses of other carnivores (Fig. 2). While marking by foxes was anecdotal for other carcasses (10%), foxes marked 40–74% of fox carcasses (Fig. 2). At conspecific carcass sites, rubbing by foxes was less frequent than marking in all study areas, while the opposite was true for heterospecific carcass sites (Table 1, Fig. 2).

According to the GLMs, with regards to fox carcasses in the three study areas, the time elapsed between carcass detection and contact by foxes was mostly related to habitat (Table 2), with foxes contacting carcasses sooner in open habitats (Table 3). Regarding carcasses of fox and other carnivores in Espuña, the time of first contact by foxes was mainly dependent on carcass type (Table 2), with foxes contacting heterospecific earlier than conspecific carcasses (Table 3); hour of carcass placement and carcass detection time by foxes also had an influence (Tables 2 and 3). However, selected models explained little of the variability in the response variable, as revealed by their low D^2^ values (< 11%; Table 2), which indicates that fox behavior was mostly conditioned by other variables not taken into account in this study.

**Table 2:** AICc-based model selection to assess the factors influencing “time of first contact” by foxes of mesocarnivore carcasses in three study areas of southeastern Spain (“among areas” comparisons) and on conspecific and heterospecific carcasses in one of these study areas (“fox *vs*. other carcasses” comparisons)

Comparison	Model	k	AICc	ΔAICc	D^2^
Among areas (fox carcasses)	**Habitat**	**1**	**342.58**	**0**	**7.89**
	Detection time	1	346.22	3.64	
	Season	1	346.53	3.95	
	Hour	1	346.57	3.99	
	Year	2	347.21	4.63	
	Area	2	347.97	5.39	
Fox *vs*. other carcasses	**Carcass**	**1**	**113.35**	**0**	**10.79**
	**Detection time**	**1**	**114.21**	**0.86**	**5.50**
	**Hour**	**1**	**114.29**	**0.94**	**5.04**

Explanatory variables include study “area”, “habitat”, “year”, “season”, “hour”, and “carcass type” (see main text for details on the variables). Number of estimated parameters (k), AICc values, AICc differences (ΔAICc) with the model with the lowest AICc, and the variability of the models explained by the predictors (deviance, D^2^) are shown. Selected models are in bold

**Table 3:** Generalized linear models (GLMs) showing the relationship between “time of first contact” by foxes with the explanatory variables included in the selected models (“habitat”: open, close; “carcass” type: fox, other; “detection time”: carcass detection time by foxes; “hour”: morning, afternoon)

Comparison	Model	Parameter	Estimate	SE	df
Among areas (fox carcasses)	Habitat	Intercept	11.40	1.89	44
		Habitat (open)	-6.67	3.27	
Fox *vs*. other carcasses	Carcass	Intercept	12.38	3.53	14
		Carcass (other)	-5.72	4.56	
	Detection time	Intercept	11.21	3.48	14
		Detection time	-0.30	0.34	
	Hour	Intercept	9.69	2.48	14
		Hour (morning)	-5.63	6.78	

Only selected models are shown, ordered from highest to lowest D^2^. The estimate of the parameters (including the sign), the standard error of the parameters (SE) and the degree of freedom of the models (df) are shown

### Fox behavior in relation to distance to carcass

**Fig. 3:**
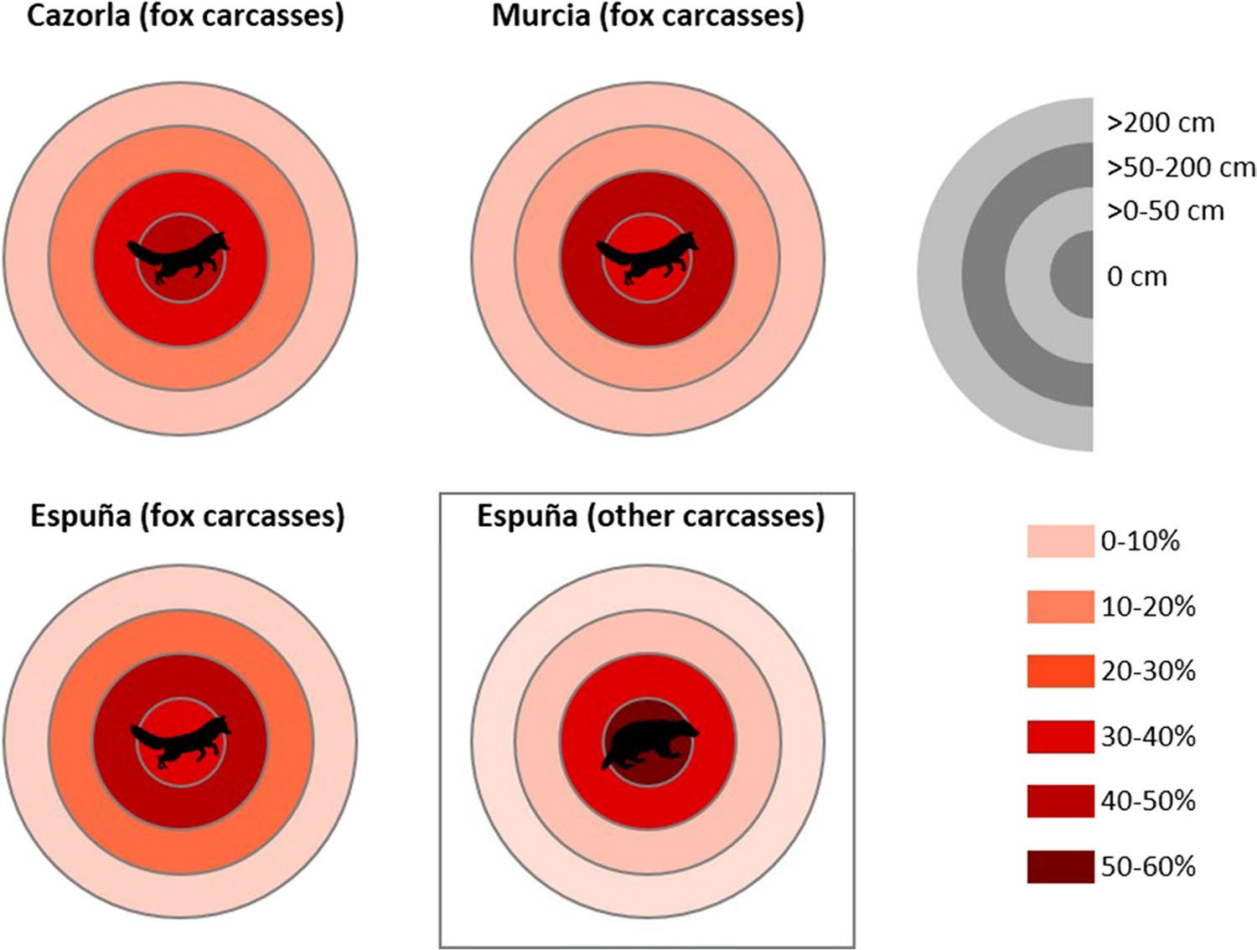
Minimum distance between visiting foxes and carcasses per study area and carcass type. Percentages are based on total events recorded per carcass type and study area

Most of the recorded events involving foxes occurred close to the carcasses (Fig. 3). The average distance between foxes and conspecific carcasses was similar in the three study areas (Cazorla and Murcia: χ^2^ = 1.603, d.f. = 3, p = 0.7; Cazorla and Espuña: χ^2^ = 4.792, d.f. = 2, p = 0.09; Murcia and Espuña: χ^2^ = 1.939, d.f. = 2, p = 0.4). However, we observed differences between carcass types: within Espuña, we recorded more fox events close to heterospecific carcasses than to conspecific ones (χ^2^ = 16.392, d.f. = 2, p < 0.001; Fig. 3).

## Discussion

We carried out a detailed behavioral study of carnivore vertebrates, including scavenging and non-scavenging species, at mammalian carnivore carcass sites, which may represent hotspots for non-trophically transmitted pathogens. To date, few studies have addressed how perceived risk of acquiring pathogens shapes the landscape of disgust of animals (Buck et al. 2018; Weinstein et al. 2018a), and none of these studies have focused on non-trophically transmitted pathogens related to carnivore carcasses (Moleón and Sánchez-Zapata 2021). Thus, patterns arising from our study may provide a basis for a more accurate interpretation of the ecological aspects that characterize non-trophically transmitted pathogens in the wild (Polley and Thompson 2015), which is especially relevant in a global context of zoonotic diseases (Evans et al. 2020; Leroy et al. 2020; Tiwari et al. 2020).

### Visitor behavior at carnivore carcass sites

Carnivore carcass sites were visited by a rich community of vertebrates, though their behavior differed widely among species groups, study areas and carcass types. The long persistence of mesocarnivore carcasses in the environment due to their relatively low consumption rate (Moleón et al. 2017; Muñoz-Lozano et al. 2019) probably favored the visiting of numerous species during the long decomposition period, which lasted up to ten weeks. Contact between the visitor and the carcass was frequently recorded. However, direct contact between two different visitor species was hardly ever recorded, and it was never observed between mammals. This contrasts with herbivore carcasses, in which mammalian scavengers may have more opportunities for contact (Borchering et al. 2017), especially in the absence of competition with vultures (Ogada et al. 2012). In carnivore carcasses, visits of mammals are more spaced than in herbivore carcasses, where many scavengers can gather in the short interval during which meat is available. Thus, at carnivore carcass sites, infection risk may take place mainly for visitor-carcass contact rather than direct contact between visitors.

Visitor behavior at carcass sites was highly dependent on the scavenging habits of the species. In our study, scavenging species were responsible for most contact events (53.1–96.5%, depending on the study area; see Table S2). Contacts by non-scavengers were mainly by small passerine birds that were observed taking hair from carcasses for nest construction (Moleón and Sánchez-Zapata 2016; Moleón et al. 2017; authors’ pers. observ.; note that these species can also scavenge occasionally and prey on necrophagous insects; Moreno-Opo and Margalida 2013). Herbivores avoid carcass sites because they pose a higher risk of being attacked by scavenging predators (Cortés-Avizanda et al. 2009; Moleón and Sánchez-Zapata 2021), so carnivore carcasses should represent a low infection risk for these species in the short-term. In the mid- and long-term, however, the vegetation that vigorously grows around carcass sites (Barton et al. 2013) may attract herbivores and, consequently, may increase the risk of infection by certain soil-borne pathogens with persistent infective stages in the environment (Johnson and Thieltges 2010; Turner et al. 2014), such as eggs of *Taenia* spp., a cestode genus that includes several species of parasites whose intermediate and definitive hosts are ungulates and mammalian carnivores, respectively (Lesniak et al. 2017). Nevertheless, vegetation responses are probably weak for relatively small carcasses such as those of mesocarnivores (Teurlings et al. 2020).

Marking and rubbing behaviors were only observed for mammal visitors. Scent-marking is very frequent in carnivores and many other mammals for interspecific and, mostly, intraspecific communication. Odors derived from marking with urine, saliva or feces are not only important for territory delimitation and defense (Ralls 1971; Johnson 1973; Sillero-Zubiri and Macdonald 1998), but also play a prominent role in assessing the health status of conspecifics in many mammalian species (Poirotte et al. 2017; Kavaliers and Choleris 2018; Kavaliers et al. 2020). The frequent marking behavior observed also suggests that carnivore carcass sites may concentrate more persistent infective stages excreted by urine or feces from the host than in the surrounding landscape. This is the case, for example, for canine parvovirus (Miranda et al. 2017), canine distemper virus (Beineke et al. 2015), *Leptospira* spp. (Millán et al. 2019) and ascarids (Okulewicz et al. 2012). Marking events may also increase the attractant effect of carcass sites for both conspecifics and heterospecifics, favoring a positive feedback loop that could promote inter- and intraspecific transmission of pathogens at carcass sites (Banks et al. 2016). All of this evidence indicates the need for further research on the effect that marking a carcass site may have, not only on animal behavior, but also on the transmission and maintenance of pathogens in the wild.

Rubbing, or scent-rubbing, is also very frequent in mammals such as carnivores, though the eco-evolutionary significance of this behavior is far from clear (Rieger 1979; Gosling and McKay 1990). In our study, direct contact with the carcass was much more frequent in rubbing events than in marking ones, which suggests that the risk of acquiring multi-host pathogens transmitted directly through non-trophic mechanisms, such as *S. scabiei* (Arlian et al. 1989; Kołodziej-Sobocińska et al. 2014) or ticks (Hofmeester et al. 2018), is higher for wild canids, mustelids and viverrids that display rubbing behavior. In addition to ectoparasites, as the carcass decays, diverse endoparasite infective stages can spread around the carcass, such as *Toxocara canis* eggs, an intestinal nematode transmitted by fecal–oral route that affects domestic and wild canids (Roddie et al. 2008). Thus, touching, rubbing against the carcass or sniffing it can also be a route of contagion for this and other directly transmitted endoparasites.

Domestic species, represented by livestock (goats and sheep) and pets (dogs and cats), were recorded in a very low proportion of total and contact events, even for the most anthropized area (Murcia). This suggests that carnivore carcasses are not important hotspots of pathogen transmission for these species, at least in our study areas. There is general concern for rabies circulation among dogs, other domestic animals, wildlife and humans in several parts of the world (Hughes and Macdonald 2013; Nadin-Davis et al. 2021), though there are no cases of rabies in our study areas. However, these interactions must be considered to study other pathogens with high epidemiological relevance at the wildlife-domestic-human interface, such as SARS-CoV-2, which is characterized by rapid spread and interspecies-jumping capacity (Leroy et al. 2020). Further studies should be promoted in regions where potential contact between wildlife and domestic animals is higher.

### Fox behavior in relation to carcass type

We found important behavioral differences of red foxes at conspecific and heterospecific carcasses in Espuña. Foxes contacted heterospecific carcasses more frequently and earlier than conspecific ones, as confirmed by the GLMs, and close contact was more frequently observed at heterospecific carcasses than at fox carcasses. Similarly, rubbing by foxes was more frequent at heterospecific than conspecific carcass sites in Espuña. All of this is in accordance with the hypotheses that, in general, infection risk is higher for phylogenetically related species (Huang et al. 2014), and that carnivores avoid feeding upon conspecific carcasses because the risk of acquiring species-specific meat-borne pathogens is at a maximum (Hart 2011; Moleón et al. 2017). In the case of sarcoptic mange, the observed fox's greater reluctance to contact conspecific carrion is consistent with the fact that canids have a higher susceptibility to sarcoptic mange than other mesocarnivore species (Astorga et al. 2018; Niedringhaus et al. 2019). In this sense, it has been suggested that *S. scabiei* causes alterations in the skin microbiome and, consequently, changes in skin odor (Nimmervoll et al. 2013; DeCandia et al. 2019), which could be conditioning the elusive behavior of visitors at infected animal carcass sites, although this requires further investigation. It should be noted that carcasses used in our study belonged to healthy animals that presented a good body condition and no skin lesions compatible with sarcoptic mange. However, in the initial stages of the disease, mangy animals do not present evident lesions, which suggests that even carrion that does not have sarcoptic lesions may be infectious to the host that contacts it.

The behavior of contacting carcasses peaked several weeks after carcass deployment, especially for conspecific carcasses. Off-host survival of ectoparasites such as mites and lice decrease with time after the host dies, with survival being affected by environmental temperature and humidity (Arlian et al. 1984, 1989; Pérez-Jiménez et al. 1990). In our Mediterranean study areas, characterized by mild and dry environmental conditions, off-host survival of ectoparasites and other pathogens is probably lower than in colder and more humid environments. Foxes visiting carcasses seemed to avoid contacting them during the period of maximum risk of acquiring ectoparasites, i.e., the first weeks after the carcass was available. However, other infective stages such as ascarid eggs, some viruses or spore-forming bacteria may survive for longer periods in the carcass vicinities (Turner et al. 2014; Beineke et al. 2015; Holland 2017; Miranda et al. 2017). In this case, the strategy of foxes to delay the propensity to contact carcasses would be ineffective to avoid infection risk. Moreover, the time elapsed in detecting carcasses was usually less than a week. From an epidemiological point of view, this indicates that, even if there is no direct contact with the carcass, there is still a risk of acquiring ectoparasites, especially in the case of those with greater mobility and capacity to leave the carcass, such as fleas and ticks (Domínguez 2004; Perrucci et al. 2016). These ectoparasites are detached from the body within a few hours after host death (Nelder and Reeves 2005), remaining around the carcass while waiting for a new host. Therefore, mesocarnivore carcass sites could be considered as an epidemiological factor influencing the transmission of vector-borne pathogens, including those with zoonotic implications (Marié et al. 2012; Millán et al. 2016; Hofmeester et al. 2018).

Fox marking behavior was also conditioned by carcass type, as urination and defecation were more frequent for conspecific carcasses. This behavior does not entail, a priori, a direct contact with the carcass, so the risk of acquiring some pathogens that are usually transmitted by direct contact and have reduced mobility outside the host, such as lice and especially *S. scabiei*, is greatly reduced (Millán et al. 2016). This also suggests that marking behavior of the red fox is weakly inhibited by the infection risk associated with the presence of carcasses. In mammalian carnivores, marking is mainly associated with intraspecific communication (e.g. Sillero-Zubiri and Macdonald 1998). However, why foxes marked more conspecific than heterospecific carcasses is unclear. A possible explanation could be that fox carcasses are more attractive as long-term marking points than carcasses of other mesocarnivores. This is because the persistence of fox carcasses in the environment is higher than that of other mesocarnivore carcasses, as foxes are more prone to feed upon heterospecific carrion (Moleón et al. 2017; Muñoz-Lozano et al. 2019).

## Conclusions

Here, we disentangled the behavior of animals visiting mesocarnivore carcass sites, which may have important implications not only for understanding the epidemiology of non-trophically transmitted parasites, but also in eco-evolutionary terms. Contact events between scavengers and carcasses were far more frequent than consumption events (Moleón et al. 2017; Muñoz-Lozano et al. 2019; Gonzálvez 2020), suggesting that scavenger behavior is more constrained by the transmission risk of meat-borne parasites than the risk of acquiring non-trophically transmitted parasites. In short, the main finding of this study was the description of different behavioral patterns of visitors at mesocarnivore carcass sites, which could be considered as epidemiological key factors in future investigations to assess the risk of infection by non-trophically transmitted parasites in the wild. Moreover, this study contributes to filling major gaps in the empirical knowledge of the role of carrion in the landscape of disgust (Moleón and Sánchez-Zapata 2021), and shows the promising and varied opportunities of studying animal behavior associated with carrion resources. The impact that emerging and re-emerging diseases associated with wildlife are having on modern societies makes it necessary to conduct these types of studies, providing scientific evidence to improve our understanding of the epidemiological factors that occur in the wild.

## Supplementary Information

Below is the link to the electronic supplementary material.

Supplementary file1 (DOCX 71 KB)

## Data Availability

The datasets generated during and/or analysed during the current study are available from the corresponding author on reasonable request.
