# Supplementary material for: Understanding potential implications for non-trophic parasite transmission based on vertebrate behavior at mesocarnivore carcass sites
Source: Vet Res Commun. 2021 Jun 26;45(4):261–75. doi: 10.1007/s11259-021-09806-2 (PMC8235911; doi:10.1007/s11259-021-09806-2)
Supplement: Supplementary file 1 — Supplementary file1 (DOCX 71 KB) [file 11259_2021_9806_MOESM1_ESM.docx]

Veterinary Research Communications

Supplementary files for

**Understanding potential implications for non-trophic parasite transmission based on vertebrate behavior at mesocarnivore carcass sites**

Moisés Gonzálvez^1^, Carlos Martínez-Carrasco^1*^, Marcos Moleón^2^

^1^Dept. of Animal Health, Faculty of Veterinary Sciences, Regional Campus of International Excellence “Campus Mare Nostrum”, University of Murcia, 30100 Murcia, Spain. ^2^Dept. of Zoology, Faculty of Sciences, University of Granada, 18071 Granada, Spain.

*Corresponding author: cmcpleit@um.es

This material includes:

**Table S1.** Vertebrate species richness recorded at carcasses, per study area and carcass type, according to different vertebrate species groups and behavior.

**Table S2a.** Carcass use patterns by vertebrate species in Cazorla (fox carcasses).

**Table S2b.** Carcass use patterns by vertebrate species in Murcia (fox carcasses).

**Table S2c.** Carcass use patterns by vertebrate species in Espuña (fox carcasses).

**Table S2d.** Carcass use patterns by vertebrate species in Espuña (other carcasses).

**Table S1.** Vertebrate species richness recorded at carcasses, per study area and carcass type, according to different vertebrate species groups and behavior. Number of monitored carcasses is indicated for each study area and carcass type. Mean number of species±SD is shown. We considered carcasses contacted, marked and rubbed as those carcasses with at least one event with contact, marking or rubbing by a given scavenger group.

| **Area** | **Carcass type** | **N** | **Group** | **Visit** | **Contact** | **Marking** | **Rubbing** |
| --- | --- | --- | --- | --- | --- | --- | --- |
| Cazorla | Foxes | 27 | Red fox | 1.0±0 | 1.0±0 | 0.7±0.5 | 0.3±0.5 |
|  |  |  | Other mammals | 4.9±1.7 | 1.9±1.4 | 0.3±0.5 | 0.3±0.6 |
|  |  |  | Birds | 1.9±1.9 | 1.1±1.1 | 0 | 0 |
|  |  |  | Total | 7.8±2.7 | 4.0±1.9 | 1.1±0.7 | 0.7±0.7 |
| Murcia | Foxes | 19 | Red fox | 0.9±0.4 | 0.6±0.5 | 0.5±0.5 | 0.2±0.4 |
|  |  |  | Other mammals | 1.8±1.3 | 0.9±1.1 | 0 | 0 |
|  |  |  | Birds | 1.6±1.3 | 0.7±0.9 | 0 | 0 |
|  |  |  | Total | 4.2±2.3 | 2.2±1.7 | 0.5±0.5 | 0.2±0.4 |
| Espuña | Foxes | 10 | Red fox | 0.9±0.3 | 0.6±0.5 | 0.4±0.5 | 0.1±0.3 |
|  |  |  | Other mammals | 1.7±1.1 | 0.6±0.5 | 0.2±0.4 | 0.1±0.3 |
|  |  |  | Birds | 1.2±0.9 | 0.8±0.9 | 0 | 0 |
|  |  |  | Total | 3.8±1.9 | 2.0±1.2 | 0.6±0.7 | 0.2±0.4 |
|  | Other | 10 | Red fox | 1.0±0 | 0.9±0.3 | 0.1±0.3 | 0.2±0.4 |
|  |  |  | Other mammals | 2.2±1.1 | 1.4±0.9 | 0.5±0.5 | 0.2±0.4 |
|  |  |  | Birds | 1.5±1.2 | 0.7±0.8 | 0 | 0 |
|  |  |  | Total | 4.7±1.8 | 3.0±1.2 | 0.6±0.5 | 0.4±0.7 |

**Table S2a.** Carcass use patterns by vertebrate species in Cazorla (fox carcasses). Mean number of individuals±SD per carcass is shown, together with the total number of events with contact, marking and rubbing behaviour for each species recorded. Species that scavenge frequently in our study areas are indicated by an asterisk.

| **Class** | **Common name** | **Scientific name** | **Individuals** | **Contact** | **Marking** | **Rubbing** |
| --- | --- | --- | --- | --- | --- | --- |
| Birds | Golden eagle* | *Aquila chrysaetos* | 1.0±0 | 8 | 0 | 0 |
|  | Griffon vulture* | *Gyps fulvus* | 20.0±0 | 1 | 0 | 0 |
|  | Common raven* | *Corvus corax* | 1.6±0.6 | 16 | 0 | 0 |
|  | Carrion crow* | *Corvus corone* | 1.6±0.8 | 96 | 0 | 0 |
|  | Eurasian jay* | *Garrulus glandarius* | 1.1±0.2 | 64 | 0 | 0 |
|  | Little owl | *Athene noctua* | 1.0±0 | 0 | 0 | 0 |
|  | Red-legged partridge | *Alectoris rufa* | - | - | - | - |
|  | European greenfinch | *Chloris chloris* | - | - | - | - |
|  | Common chaffinch | *Fringilla coelebs* | 2.0±0 | 0 | 0 | 0 |
|  | Blue tit | *Cyanistes caeruleus* | 1.0±0 | 0 | 0 | 0 |
|  | Great tit | *Parus major* | 1.7±1.9 | 4 | 0 | 0 |
|  | Black redstart | *Phoenicurus ochruros* | 1.0±0 | 1 | 0 | 0 |
|  | European robin | *Erithacus rubecula* | 1.0±0 | 10 | 0 | 0 |
|  | Rock dove | *Columba palumbus* | 1.0±0 | 0 | 0 | 0 |
|  | Woodchat shrike | *Lanius senator* | - | - | - | - |
|  | Wood lark | *Lullula arborea* | 1.0±0.2 | 1 | 0 | 0 |
|  | Blackcap | *Sylvia atricapilla* | - | - | - | - |
|  | Common blackbird | *Turdus merula* | 1.0±0 | 0 | 0 | 0 |
|  | Hoopoe | *Upupa epops* | - | - | - | - |
| Mammals | Barbary sheep | *Ammotragus lervia* | - | - | - | - |
|  | Goat | *Capra aegagrus hircus* | - | - | - | - |
|  | Spanish ibex | *Capra pyrenaica* | 1.4±0.8 | 1 | 0 | 0 |
|  | Sheep | *Ovis aries* | 9.1±10.2 | 1 | 0 | 0 |
|  | Mouflon | *Ovis musimon* | 1.6±0.8 | 1 | 0 | 0 |
|  | Red deer | *Cervus elaphus* | 1.2±0.4 | 0 | 0 | 0 |
|  | Fallow deer | *Dama dama* | 1.4±0.7 | 1 | 0 | 0 |
|  | Wild boar* | *Sus scrofa* | 1.3±0.9 | 79 | 0 | 12 |
|  | Dog* | *Canis lupus familiaris* | 1.3±0.5 | 9 | 0 | 2 |
|  | Red fox* | *Vulpes vulpes* | 1.0±0.1 | 275 | 76 | 20 |
|  | Domestic cat | *Felis silvestris catus* | - | - | - | - |
|  | Wildcat | *Felis silvestris silvestris* | 1.0±0 | 1 | 0 | 0 |
|  | Common genet* | *Genetta genetta* | 1.0±0 | 16 | 1 | 0 |
|  | Stone marten* | *Martes foina* | 1.0±0 | 36 | 11 | 0 |
|  | Eurasian badger* | *Meles meles* | 1.0±0 | 0 | 0 | 0 |
|  | Long-tailed field mouse* | *Apodemus sylvaticus* | - | - | - | - |
|  | Rat | *Rattus* spp*.* | 1.0±0 | 1 | 0 | 0 |
|  | Garden dormouse* | *Eliomys quercinus* | 1.0±0 | 0 | 0 | 0 |
|  | Red squirrel | *Sciurus vulgaris* | 1.1±0.3 | 1 | 0 | 0 |
|  | European rabbit | *Oryctolagus cuniculus* | - | - | - | - |
|  | White-toothed shrew | *Crocidura russula* | - | - | - | - |
| Reptiles | Ocellated lizard* | *Timon lepidus* | - | - | - | - |
| **Total** |  |  |  | **623** | **88** | **34** |

**Table S2b.** Carcass use patterns by vertebrate species in Murcia (fox carcasses). Mean number of individuals±SD per carcass is shown, together with the total number of events with contact, marking and rubbing behaviour for each species recorded. Species that scavenge frequently in our study areas are indicated by an asterisk.

| **Class** | **Common name** | **Scientific name** | **Individuals** | **Contact** | **Marking** | **Rubbing** |
| --- | --- | --- | --- | --- | --- | --- |
| Birds | Golden eagle* | *Aquila chrysaetos* | - | - | - | - |
|  | Griffon vulture* | *Gyps fulvus* | - | - | - | - |
|  | Common raven* | *Corvus corax* | - | - | - | - |
|  | Carrion crow* | *Corvus corone* | - | - | - | - |
|  | Eurasian jay* | *Garrulus glandarius* | - | - | - | - |
|  | Little owl | *Athene noctua* | 1.0±0 | 6 | 0 | 0 |
|  | Red-legged partridge | *Alectoris rufa* | 1.5±1.2 | 0 | 0 | 0 |
|  | European greenfinch | *Chloris chloris* | 1.0±0 | 0 | 0 | 0 |
|  | Common chaffinch | *Fringilla coelebs* | - | - | - | - |
|  | Blue tit | *Cyanistes caeruleus* | - | - | - | - |
|  | Great tit | *Parus major* | - | - | - | - |
|  | Black redstart | *Phoenicurus ochruros* | 1.1±0.3 | 4 | 0 | 0 |
|  | European robin | *Erithacus rubecula* | 1.0±0.1 | 19 | 0 | 0 |
|  | Rock dove | *Columba palumbus* | - | - | - | - |
|  | Woodchat shrike | *Lanius senator* | 1.0±0 | 1 | 0 | 0 |
|  | Wood lark | *Lullula arborea* | 1.0±0.2 | 0 | 0 | 0 |
|  | Blackcap | *Sylvia atricapilla* | 1.0±0 | 1 | 0 | 0 |
|  | Common blackbird | *Turdus merula* | 1.0±0 | 0 | 0 | 0 |
|  | Hoopoe | *Upupa epops* | 1.0±0 | 0 | 0 | 0 |
| Mammals | Barbary sheep | *Ammotragus lervia* | - | - | - | - |
|  | Goat | *Capra aegagrus hircus* | 2.0±0 | 0 | 0 | 0 |
|  | Spanish ibex | *Capra pyrenaica* | - | - | - | - |
|  | Sheep | *Ovis aries* | - | - | - | - |
|  | Mouflon | *Ovis musimon* | - | - | - | - |
|  | Red deer | *Cervus elaphus* | - | - | - | - |
|  | Fallow deer | *Dama dama* | - | - | - | - |
|  | Wild boar* | *Sus scrofa* | 1.7±1.9 | 7 | 0 | 0 |
|  | Dog* | *Canis lupus familiaris* | 1.1±0.4 | 7 | 0 | 0 |
|  | Red fox* | *Vulpes vulpes* | 1.0±0.7 | 62 | 22 | 11 |
|  | Domestic cat | *Felis silvestris catus* | 1.0±0 | 0 | 0 | 0 |
|  | Wildcat | *Felis silvestris silvestris* | 1.0±0 | 1 | 0 | 0 |
|  | Common genet* | *Genetta genetta* | 1.0±0 | 2 | 0 | 0 |
|  | Stone marten* | *Martes foina* | 1.0±0 | 0 | 0 | 0 |
|  | Eurasian badger* | *Meles meles* | - | - | - | - |
|  | Long-tailed field mouse* | *Apodemus sylvaticus* | 1.0±0 | 5 | 0 | 0 |
|  | Rat | *Rattus* spp*.* | - | - | - | - |
|  | Garden dormouse* | *Eliomys quercinus* | 1.0±0 | 39 | 0 | 0 |
|  | Red squirrel | *Sciurus vulgaris* | 1.0±0 | 1 | 0 | 0 |
|  | European rabbit | *Oryctolagus cuniculus* | 1.0±0 | 1 | 0 | 0 |
|  | White-toothed shrew | *Crocidura russula* | 1.0±0 | 4 | 0 | 0 |
| Reptiles | Ocellated lizard* | *Timon lepidus* | - | - | - | - |
| **Total** |  |  |  | **160** | **22** | **11** |

**Table S2c.** Carcass use patterns by vertebrate species in Espuña (fox carcasses). Mean number of individuals±SD per carcass is shown, together with the total number of events with contact, marking and rubbing behaviour for each species recorded. Species that scavenge frequently in our study areas are indicated by an asterisk.

| **Class** | **Common name** | **Scientific name** | **Individuals** | **Contact** | **Marking** | **Rubbing** |
| --- | --- | --- | --- | --- | --- | --- |
| Birds | Golden eagle* | *Aquila chrysaetos* | - | - | - | - |
|  | Griffon vulture* | *Gyps fulvus* | - | - | - | - |
|  | Common raven* | *Corvus corax* | - | - | - | - |
|  | Carrion crow* | *Corvus corone* | - | - | - | - |
|  | Eurasian jay* | *Garrulus glandarius* | 1.2±0.4 | 13 | 0 | 0 |
|  | Little owl | *Athene noctua* | - | - | - | - |
|  | Red-legged partridge | *Alectoris rufa* | - | - | - | - |
|  | European greenfinch | *Chloris chloris* | - | - | - | - |
|  | Common chaffinch | *Fringilla coelebs* | - | - | - | - |
|  | Blue tit | *Cyanistes caeruleus* | - | - | - | - |
|  | Great tit | *Parus major* | 1.1±0.3 | 26 | 0 | 0 |
|  | Black redstart | *Phoenicurus ochruros* | - | - | - | - |
|  | European robin | *Erithacus rubecula* | 1.0±0 | 0 | 0 | 0 |
|  | Rock dove | *Columba palumbus* | - | - | - | - |
|  | Woodchat shrike | *Lanius senator* | - | - | - | - |
|  | Wood lark | *Lullula arborea* | - | - | - | - |
|  | Blackcap | *Sylvia atricapilla* | - | - | - | - |
|  | Common blackbird | *Turdus merula* | 1.5±1.0 | 0 | 0 | 0 |
|  | Hoopoe | *Upupa epops* | - | - | - | - |
| Mammals | Barbary sheep | *Ammotragus lervia* | 1.7±1.2 | 1 | 0 | 0 |
|  | Goat | *Capra aegagrus hircus* | - | - | - | - |
|  | Spanish ibex | *Capra pyrenaica* | - | - | - | - |
|  | Sheep | *Ovis aries* | - | - | - | - |
|  | Mouflon | *Ovis musimon* | - | - | - | - |
|  | Red deer | *Cervus elaphus* | - | - | - | - |
|  | Fallow deer | *Dama dama* | - | - | - | - |
|  | Wild boar* | *Sus scrofa* | 2.3±3.5 | 5 | 1 | 1 |
|  | Dog* | *Canis lupus familiaris* | - | - | - | - |
|  | Red fox* | *Vulpes vulpes* | 1.0±0 | 24 | 9 | 4 |
|  | Domestic cat | *Felis silvestris catus* | - | - | - | - |
|  | Wildcat | *Felis silvestris silvestris* | - | - | - | - |
|  | Common genet* | *Genetta genetta* | 1.0±0 | 0 | 1 | 0 |
|  | Stone marten* | *Martes foina* | 1.0±0 | 1 | 0 | 0 |
|  | Eurasian badger* | *Meles meles* | - | - | - | - |
|  | Long-tailed field mouse* | *Apodemus sylvaticus* | - | - | - | - |
|  | Rat | *Rattus* spp*.* | - | - | - | - |
|  | Garden dormouse* | *Eliomys quercinus* | - | - | - | - |
|  | Red squirrel | *Sciurus vulgaris* | 2.0±1.7 | 0 | 0 | 0 |
|  | European rabbit | *Oryctolagus cuniculus* | - | - | - | - |
|  | White-toothed shrew | *Crocidura russula* | - | - | - | - |
| Reptiles | Ocellated lizard* | *Timon lepidus* | 1.0±0 | 4 | 0 | 0 |
| **Total** |  |  |  | **74** | **11** | **5** |

**Table S2d.** Carcass use patterns by vertebrate species in Espuña (other carcasses). Mean number of individuals±SD per carcass is shown, together with the total number of events with contact, marking and rubbing behaviour for each species recorded. Species that scavenge frequently in our study areas are indicated by an asterisk.

| **Class** | **Common name** | **Scientific name** | **Individuals** | **Contact** | **Marking** | **Rubbing** |
| --- | --- | --- | --- | --- | --- | --- |
| Birds | Golden eagle* | *Aquila chrysaetos* | - | - | - | - |
|  | Griffon vulture* | *Gyps fulvus* | - | - | - | - |
|  | Common raven* | *Corvus corax* | - | - | - | - |
|  | Carrion crow* | *Corvus corone* | - | - | - | - |
|  | Eurasian jay* | *Garrulus glandarius* | 1.0±0 | 3 | 0 | 0 |
|  | Little owl | *Athene noctua* | - | - | - | - |
|  | Red-legged partridge | *Alectoris rufa* | 1.5±0.7 | 0 | 0 | 0 |
|  | European greenfinch | *Chloris chloris* | - | - | - | - |
|  | Common chaffinch | *Fringilla coelebs* | - | - | - | - |
|  | Blue tit | *Cyanistes caeruleus* | - | - | - | - |
|  | Great tit | *Parus major* | 1.1±0.3 | 13 | 0 | 0 |
|  | Black redstart | *Phoenicurus ochruros* | - | - | - | - |
|  | European robin | *Erithacus rubecula* | - | - | - | - |
|  | Rock dove | *Columba palumbus* | - | - | - | - |
|  | Woodchat shrike | *Lanius senator* | - | - | - | - |
|  | Wood lark | *Lullula arborea* | 1.0±0 | 0 | 0 | 0 |
|  | Blackcap | *Sylvia atricapilla* | - | - | - | - |
|  | Common blackbird | *Turdus merula* | 1.0±0 | 0 | 0 | 0 |
|  | Hoopoe | *Upupa epops* | - | - | - | - |
| Mammals | Barbary sheep | *Ammotragus lervia* | 1.3±0.5 | 0 | 0 | 0 |
|  | Goat | *Capra aegagrus hircus* | - | - | - | - |
|  | Spanish ibex | *Capra pyrenaica* | - | - | - | - |
|  | Sheep | *Ovis aries* | - | - | - | - |
|  | Mouflon | *Ovis musimon* | - | - | - | - |
|  | Red deer | *Cervus elaphus* | - | - | - | - |
|  | Fallow deer | *Dama dama* | - | - | - | - |
|  | Wild boar* | *Sus scrofa* | 1.5±1.1 | 7 | 0 | 0 |
|  | Dog* | *Canis lupus familiaris* | 1.5±0.7 | 2 | 0 | 2 |
|  | Red fox* | *Vulpes vulpes* | 1.0±0.1 | 75 | 6 | 5 |
|  | Domestic cat | *Felis silvestris catus* | - | - | - | - |
|  | Wildcat | *Felis silvestris silvestris* | - | - | - | - |
|  | Common genet* | *Genetta genetta* | 1.0±0 | 1 | 1 | 0 |
|  | Stone marten* | *Martes foina* | 1.0±0 | 5 | 8 | 0 |
|  | Eurasian badger* | *Meles meles* | - | - | - | - |
|  | Long-tailed field mouse* | *Apodemus sylvaticus* | - | - | - | - |
|  | Rat | *Rattus* spp*.* | 1.0±0 | 1 | 0 | 0 |
|  | Garden dormouse* | *Eliomys quercinus* | 1.0±0 | 3 | 0 | 0 |
|  | Red squirrel | *Sciurus vulgaris* | 1.0±0 | 0 | 0 | 0 |
|  | European rabbit | *Oryctolagus cuniculus* | 1.0±0 | 0 | 0 | 0 |
|  | White-toothed shrew | *Crocidura russula* | - | - | - | - |
| Reptiles | Ocellated lizard* | *Timon lepidus* | 1.0±0 | 1 | 0 | 0 |
| **Total** |  |  |  | **111** | **15** | **7** |
